# An antiarrhythmic approach to hydroxychloroquine-induced QT prolongation

**DOI:** 10.1007/s12471-020-01464-4

**Published:** 2020-07-08

**Authors:** E. Yetkin, K. Yalta, J. Waltenberger

**Affiliations:** 1 Department of Cardiology, Istinye University Faculty of Medicine, Liv Hospital, Istanbul, Turkey; 2 Department of Cardiology, Faculty of Medicine, Trakya University, Edirne, Turkey; 3 Department of Cardiovascular Medicine, University of Münster, Münster, Germany; 4 Department of Internal Medicine I, SRH Central Hospital, Suhl, Germany

Derivatives of quinolone, namely quinidine, quinine and hydroxychloroquine (HCQ, hydroxylated form of aminoquinone), have been used for decades in the treatment of different diseases, including malaria, rheumatological diseases, cardiac arrhythmias and, most recently, the new coronavirus disease 2019 (COVID-19). Although quinidine is no longer widely used for the termination and prevention of arrhythmias, quinine and HCQ are still in common use for rheumatological diseases and malaria. Their common side-effects, QT prolongation and a risk of proarrhythmia, have re-emerged as a result of the widespread use of HCQ in the treatment of COVID-19. In fact, they all show the main electrophysiological aspects of class Ia antiarrhythmic drugs. This antiarrhythmic effect is mainly characterised by the inhibition of fast Na channels and to a lesser extent by K‑channel inhibition. However, this class I antiarrhythmic effect is accompanied by a proarrhythmic effect by prolonging the QT interval, thereby facilitating the occurrence of torsades de pointes or ventricular arrhythmias. Therefore, being a derivative of quinolone, like quinidine, and manifesting class Ia antiarrhythmic drug effects, HCQ has been given all the attention regarding the treatment of COVID-19 patients. In this regard, van den Broek et al. have recently documented that chloroquine significantly prolongs the QT interval in a clinically relevant manner [1]. Chloroquine treatment resulted in a mean QTc prolongation of 35 ms (95% confidence interval (CI) 28–43 ms) using computerised interpretation and 34 ms (95% CI 25–43 ms) using manual interpretation. Although no torsades de pointes were observed during chloroquine treatment, 23% of patients had a QTc interval exceeding 500 ms during chloroquine treatment. These findings highlight the need for ECG monitoring when prescribing chloroquine to COVID-19 patients.

It should be emphasised that the increase in QT interval in COVID-19 patients receiving HCQ has been observed in the absence of concomitant use of azithromycin (AZM). HCQ + AZM could have resulted in a further increase in QT interval in those patients. We would also like to comment on a small but encouraging item in their patient cohort. Given the fact that HCQ is a member of the quina-quina family and has the electrophysiological features of quinidine, it would be valuable to know the details of concurrent antiarrhythmic use (ATC class C01B) in COVID-19 patients in terms of QT prolongation. Van den Broek et al. reported that 4% of patients had already been using antiarrhythmic drugs, ATC class C01B including class Ia, Ib, Ic and class III. Except for the class Ib antiarrhythmic drugs (mexiletine and lidocaine) all ATC class C01B drugs have the potential for QRS and/or QT prolongation. In contrast to class Ia antiarrhythmics and HCQ, class Ib antiarrhythmics have clinically relevant QT-shortening effects [2]. Therefore, it might be a reasonable approach to administer mexiletine and lidocaine in COVID-19 patients with critical QT prolongation or a QT interval exceeding 500 ms. A recent report demonstrated successful completion of HCQ + AZM therapy with shortening of the QR interval from 620 to 550 ms by concurrent use of lidocaine [3].

In this context, concurrent use of lidocaine and mexiletine might be a promising approach to completing the HCQ and/or AZM treatment in patients with critical QT prolongation or a QT interval exceeding 500 ms.
